# *Mycobacterium genavense* infection in two species of captive snakes

**DOI:** 10.1186/s40409-016-0082-7

**Published:** 2016-10-18

**Authors:** Leila Sabrina Ullmann, Ramiro das Neves Dias-Neto, Didier Quevedo Cagnini, Ricardo Seiti Yamatogi, Jose Paes Oliveira-Filho, Viviane Nemer, Rodrigo Hidalgo Friciello Teixeira, Alexander Welker Biondo, João Pessoa Araújo

**Affiliations:** 1 Department of Microbiology and Immunology, Botucatu Biosciences Institute, São Paulo State University (UNESP - Univ Estadual Paulista), Distrito de Rubião Junior, s/n, Botucatu, SP 80035-050 Brazil; 2 Sorocaba Municipal Zoo Park (Quinzinho de Barros), Sorocaba, SP Brazil; 3 Department of Clinical Veterinary Medicine, School of Veterinary Medicine and Animal Husbandry, São Paulo State University (UNESP - Univ Estadual Paulista), Botucatu, SP Brazil; 4 Veterinary Practitioner, Pathology Anatomy Specialist, Sorocaba, SP Brazil; 5 Department of Veterinary Medicine, Federal University of Paraná, Curitiba, Brazil

**Keywords:** Captive snakes, *Mycobacterium*, Public health

## Abstract

**Background:**

*Mycobacterium* is an important zoonotic agent with companion, livestock and wildlife animals reportedly playing a role as reservoirs. Although its association with reptiles has been described, the disease cycle remains to be fully established, particularly in snakes. Accordingly, this study aimed to report the occurrence of mycobacteriosis with clinical pneumonia in one exotic python snake (*Python molurus*) and one native green snake (*Philodryas olfersii*) from the Sorocaba Zoo, São Paulo state, Brazil.

**Methods:**

Diagnosis was based on necropsy, histopathological examination, Ziehl-Neelsen stain and immunohistochemistry.

**Results:**

Using a nested PCR followed by DNA sequencing and bioinformatics analysis, the causative *Mycobacterium* species was identified as *Mycobacterium genavense*.

**Conclusion:**

*Mycobacterium genavense* is an infectious zoonotic agent of animal and public health concerns.

## Background

Mycobacteriosis is a generic term for many diseases with different pathogenicities caused by about 85 bacterial species of the genus *Mycobacterium* [1]. Mycobacteria are gram-positive, aerobic, alcohol-acid-resistant bacteria and share the same morphological characteristics among different species, which are currently classified into *M. tuberculosis* and nontuberculous mycobacteria (NTM) [2, 3].

Cold-blooded animals may carry a wide variety of zoonotic pathogens; in particular, reptiles may pose a health risk to professionals with whom they have contact. Although mostly described in mammals, reptiles may also be affected by mycobacteriosis, presenting clinical signs related to the affected organs and characterized by diffuse and granulomatous lesions [4]. Diagnosis is usually postmortem, wherein necropsy findings when visible include greyish-white nodules due to the granulomatous inflammatory infiltrate with multinucleated giant cells; differently from mammals, mineralized nodules are not observed in reptiles [2, 4].

Mycobacterium infection has been reported in reptiles and attributed to several species such as *M. avium*, *M. chelonae*, *M. fortuitum*, *M. intracellulare*, *M. marinum*, *M. phlei*, *M. smegmatis* and *M. ulcerans*, with a single case of *M. tuberculosis* complex infection [2, 5, 6]. Only five cases of *Mycobacterium* infection have been described in snakes between 1928 and 2002, including the species *Boa constrictor*, *Elaphe quadrivitta* and *Python regius* [6–10]. Nonetheless, mycobacteriosis transmission has been poorly reported and remains to be fully established in reptiles [4]. Cutaneous lesions and/or ingestion may represent the main pathogenic pathways to susceptible and more likely immunosuppressed animals; impaired immune status may be triggered by chronic diseases, stress, poor nutrition and captivity [4]. Illness may be accompanied by non-specific signs such as anorexia, lethargy and weight loss, while the main lesions found in snakes with mycobacteria may include skin alteration, stomatitis, and respiratory tract infection in animals with chronic disease [11, 12].

Snakes have become a growing market as pets, sharing household spaces and increasing the owners’ risks of bacterial infection. *Mycobacterium fortuitum* and *M. fortuitum*-like were isolated from domestic reptiles, ball pythons (*Python regius*), either in private owners’ residences or in pet shops. Pet reptiles, including snakes, can act as possible reservoirs of pathogens and represent a serious public health risk to humans, in particular immune-compromised patients, and other domestic animals. Also, regular cage sanitation and personal hygiene after animal handling are important to avoid pathogen transmission [4].

Although individually maintained, captive snakes may share common handling, carrying and food, exposing immunosuppressed native snake species to exotic *Mycobacterium* spp. infection or *vice versa*. Regardless, such information may provide important data for future improvement of sanitary conditions and disease prevention among captive snake species worldwide. Accordingly, the aim of this study was to report the occurrence of mycobacteriosis in one exotic and one native snake species at the Sorocaba Zoo, São Paulo state, southeastern Brazil.

## Methods

One four-month-old male zoo-born python (*Python molurus*) and one adult female wild-caught green snake (*Philodryas olfersii*) kept at Sorocaba Zoo, São Paulo state, Brazil, were presented at the veterinary section with similar clinical signs, including lethargy, mild anorexia and weight loss. Despite immediate and intensive therapy for pneumonia, both snakes died after 2 days and were submitted to necropsy as part of zoo routinely protocol. Prior to these two cases, no other snake in the zoo had shown any clinical sign of death without known cause.

Both snake carcasses were separately kept under refrigeration until individual necropsies were performed at different times to avoid cross-contamination of samples. Representative tissue samples were collected from lung, trachea, heart, kidneys, esophagus, and small intestine, preserved in 10 % neutral buffered formalin, and submitted to histopathological examination. Unstained slides were prepared for prospective evaluation by Ziehl-Neelsen staining and immunohistochemistry (IHC). A careful search for *Mycobacterium* was performed on all tissue areas with granulomatous inflammation*.*

Tissues for IHC were sectioned (3-μm thick), deparaffinized, rehydrated, and immersed in 3 % hydrogen peroxide solution in methanol for 20 min. Antigen retrieval was performed by heat treatment in 10 mM citrate buffer, pH 6.0, for the primary antibodies. The sections were digested with proteinase K (25 μg/mL in TE buffer, pH 8.0) for 15 min at 37 °C. Non-specific binding was blocked with 3 % skim milk in phosphate-buffered saline for one hour. The primary antibody utilized was the anti-bacillus Calmette-Guérin (BCG-*Mycobacterium bovis*) by means of a standard streptavidin-biotin-peroxidase technique. A positive case of human tuberculosis was used as positive control.

Total DNA was isolated from paraffin-embedded samples using a commercial tissue kit (QIAamp DNA FFPE, Qiagen, USA) following the manufacturer’s protocol, and stored at −20 °C until processing. All handling, extraction, and amplification procedures were performed in separate laboratories using disposable tips with barrier protection. DNA purity and concentration were obtained using a commercial spectrophotometer (NanoDrop, Thermo Scienfic, USA), in which A260/280 and A260/230 values greater than 1.8 were considered suitable for analysis. The nested PCR (nPCR) was performed using a set of primers to a 16S region of ribosomal RNA of the *Mycobacterium* genus. The first-round primer sets used in nPCR, amplifying 590 base pairs (bp), were forward 246 (5′-AGAGTTTGATCCTGGCTCAG-3′) and reverse 247 (5′-TTTCACGAACAAGCCCAGAA3′). Second-round primers set (amplifying 455 bp) were forward M1 (5′-AGTGGCGAACGGGTGAGTAAC-3′) and reverse R7 (5′-TTACG CCCAGTAATTCCGGACAA-3′) [13]. The nested PCR conditions were performed as previously described [14].

The PCR products were analyzed by 1.5 % agarose gel electrophoresis and visualized using GelRed™ (Biotium, USA). The molecular weights were estimated by comparison with a known marker (100 bp ladder) and gels documented with a commercial imaging capture system (ImageQuant System, GE Healthcare, USA).

The amplified products were purified using an available commercial kit (Invisorb Fragment CleanUp, Invitrogen, USA). The sequencing reactions were performed with the ABI 3500 Sequence Detection System (Applied Biosystems, USA). The sequences homologies were verified using the software Geneious R6 (Geneious Company, New Zealand).

## Results and discussion

**Fig. 1:**
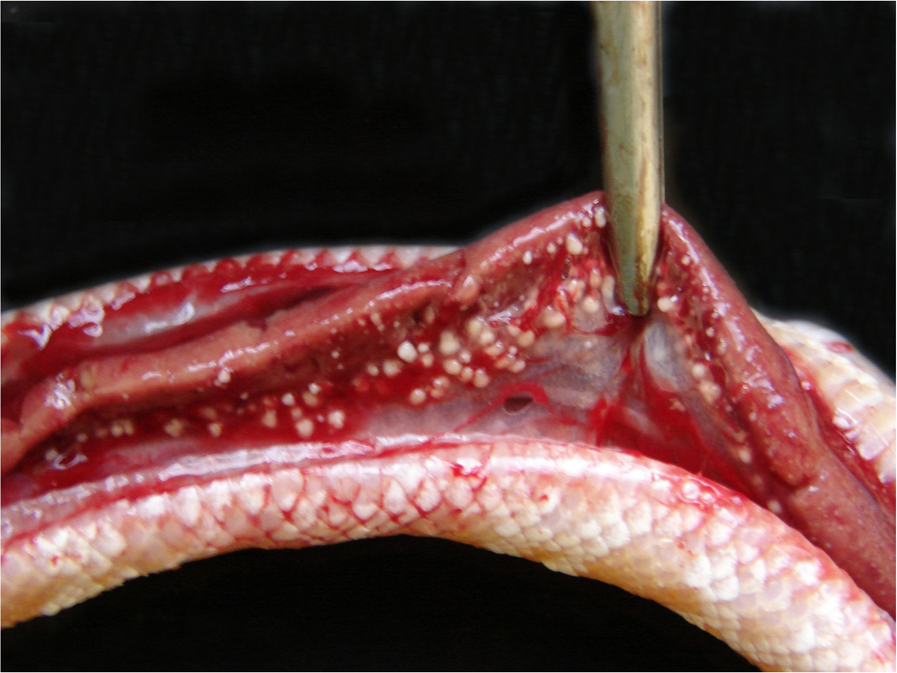
Mycobacteriosis in *Python molurus*. Liver with whitish, multifocal nodules, randomly spread, indicating a granulomatous inflammatory process

Whitish nodules a few millimeters in diameter found in the trachea, lung and liver (Fig. 1), hyperemic intestines and pale kidneys during the python necropsy examination led to suspicion of *Mycobacterium* spp. infection. Similar whitish, multifocal nodules were observed in the green snake; however, randomly spread only in lung and liver tissue.

**Fig. 2:**
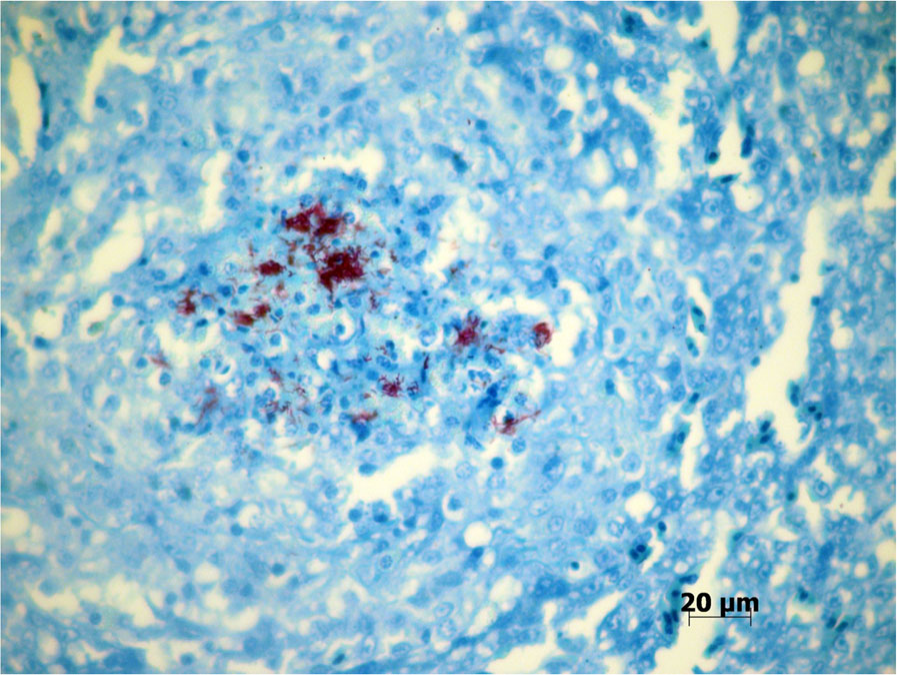
Mycobacteriosis in *Python molurus*. Lung with innumerous Ziehl-Neelsen-positive bacilli in a necrotic area (magnification 40×)

Microscopic examination of python samples revealed pneumonia (Fig. 2) which was characterized by mild heterophilic inflammatory interstitial infiltration and necrotic areas surrounded by heterophils, macrophages and giant cells. Similar lesions were found in the esophagus, trachea and liver. Mild heterophilic inflammatory infiltration was observed in the heart tissue. The green snake presented pneumonia lesions similar to those observed in the python*.*

Due to nonspecific macroscopic lesions observed at necropsy and granulomatous inflammation generally associated with infectious agents resistant to phagocytosis, mycobacterial infection was included in the differential diagnosis. Bacilli, stained positively by Ziehl-Neelsen, suggested the presence of *Mycobacterium* infection [15].

Besides the inflammatory alterations and the presence of acid-alcohol-resistant bacilli stained by Ziehl-Neelsen, samples were also positive for *Mycobacterium* genus on immunohistochemistry. As previously described, both immunohistochemistry and molecular techniques should be used in suspected *Mycobacterium* cases [1].

Nested PCR followed by DNA sequencing was applied to ascertain the particular mycobacterial species. The amplified product sequences were blasted, resulting in a 100 % match with *M. genavense* based on Linnaeus Blast tool (Geneious R6 software, Geneious, New Zealand) and NCBI nucleotide Blast. The sequences were submitted to GenBank (KJ614669 and KJ614670).

No ante-mortem test has been considered 100 % reliable for the detection of mycobacteriosis in wild animals, but the presence of *Mycobacterium* genus in snakes has been reported in live animals [1]. Bacteria of the genus *Mycobacterium* were isolated in 13/18 (72.2 %) snakes, 13/134 (9.7 %) lizards, and 11/71 (15.5 %) turtles, all clinically healthy animals bred in captivity. Mycobacteria were classified as *M. fortuitum* (14; 37.8 %), *M. fortuitum*-like (17; 45.9 %), *M. peregrinum* (4; 10.8 %), or *M. chelonae* (1; 10.8 %). *M. fortuitum* was isolated from seven pythons and *M. fortuitum*-like from six pythons, demonstrating that clinically healthy animals may harbor and transmit the agent, acting as reservoirs of public health concern to owners, handlers and veterinarians [4].

The mycobacteriosis infection was first diagnosed by positive Ziehl-Neelsen reaction (Fig. 2). As *Nocardia* bacterial infection may also be positively stained by Ziehl-Neelsen, immunohistochemistry test based on specific antigen-antibody reaction was used to confirm the presence of mycobacteria [15].

Mycobacterial infection was confirmed by the histological, histochemical, immunohistochemistry and molecular techniques. After sequencing, bioinformatics tools were able to identify *M. genavense* as the species implicated in the infection in both cases.

Snakes were kept in individual enclosures with restricted contact with other utensils or environmental sources so that aerosol was considered an unlikely source of transmission. Snakes were regularly fed healthy newborn mice maintained elsewhere, with no historical signs of disease or infection. Enclosure contamination or oral infection has been attributed as the probable source of infection but no consistent evidence was found.

The identification of *M. genavense* as the etiological agent may serve as a warning for potential zoonotic risk, since immunocompromised people are considered susceptible to infection [16, 17]. More studied should be conducted on mycobacterial infection to establish the role of reptiles as potential reservoirs for human or other animal pathogen transmission.

## Conclusions

Since mycobacteriosis is usually a chronic disease with non-specific findings and a complex diagnosis, few reports of mycobacterium infection in reptiles have been reported to date. The combination of immunological and molecular techniques provided general and specific mycobacterial diagnosis, with *M. genavense* implicated as the causative agent, usually considered an environmental bacterium identified in oral and cutaneous lesions of reptiles.
